# Reduction of heart failure guideline‐directed medication during hospitalization: prevalence, risk factors, and outcomes

**DOI:** 10.1002/ehf2.14051

**Published:** 2022-07-07

**Authors:** Victoria Palin, Michael Drozd, Ellis Garland, Anam Malik, Sam Straw, Melanie McGinlay, Alexander Simms, V. Kate Gatenby, Anshuman Sengupta, Eylem Levelt, Klaus K. Witte, Mark T. Kearney, Richard M. Cubbon

**Affiliations:** ^1^ Leeds Institute of Cardiovascular and Metabolic Medicine The University of Leeds Clarendon Way Leeds LS2 9JT UK; ^2^ Department of Cardiology Leeds Teaching Hospitals NHS Trust Leeds UK; ^3^ Medical Clinic 1 University Hospital Aachen, RWTH Aachen Germany

**Keywords:** Hospitalization, Medication, Dose, Non‐cardiovascular, Prognosis

## Abstract

**Aims:**

Optimal management of heart failure with reduced ejection fraction (HFrEF) includes titration of guideline‐directed medical therapy (GDMT) to the highest tolerated dose within the licensed range. During hospitalization, GDMT doses are often significantly altered, although it is unknown whether the cause of hospitalization influences this.

**Methods and results:**

We recruited 711 people with stable HFrEF from specialist heart failure clinics and prospectively assessed events occurring during first unplanned hospitalization. Dose changes of ACE inhibitors or angiotensin receptor blockers (ACEi/ARB), beta‐blockers, mineralocorticoid receptor antagonists, and loop diuretics were recorded during 414 hospitalizations, categorized as due to decompensated heart failure, other cardiovascular causes, infection, or other non‐cardiovascular causes. Most hospitalizations resulted in no change to GDMT. ACEi/ARB dose was reduced in 21% of hospitalizations and was more common during non‐cardiovascular hospitalization (25.4% vs. 13.9%; *P* = 0.005). ACEi/ARB dose reduction was associated with older age and lower left ventricular ejection fraction at study recruitment, and poorer renal function, lower systolic blood pressure, higher serum potassium, and less frequent care from a cardiologist during admission. People experiencing ACEi/ARB reduction had worse age‐adjusted survival after discharge, without differences in heart failure re‐hospitalization. De‐escalation of beta‐blockers occurred in 8% of hospitalizations, most often due to other non‐cardiovascular causes; this was not associated with post‐discharge survival or re‐hospitalization with heart failure.

**Conclusions:**

De‐escalation of HFrEF GDMT is more common during non‐cardiovascular hospitalization and for ACEi/ARB is associated with reduced survival. Post‐discharge care plans should include robust plans to consider re‐escalation of GDMT in these cases.

## Introduction

The prevalence of heart failure (HF) has risen substantially over recent decades, and it remains associated with high rates of hospitalization and mortality.^1^, ^2^ Medical therapies including ACE inhibitors (ACEi), angiotensin receptor blockers (ARBs), beta‐blockers, and mineralocorticoid receptor antagonists (MRAs) have been shown to improve these outcomes in randomized clinical trials recruiting patients with HF with reduced ejection fraction (HFrEF).^3^, ^4^, ^5^ In these trials, 50–60% of patients achieved optimal target doses.^6^ Clinical guidelines reflect this, with clear instructions on optimal medical therapy—termed guideline‐directed medical therapy (GDMT).^7^, ^8^ Despite this, people with HFrEF commonly do not receive GDMT at therapeutically optimal dose in routine clinical practice.^9^, ^10^ Some studies have reported that fewer than 1% of people with HFrEF simultaneously received stable target doses of ACEi/ARBs, beta‐blockers, and MRAs.^9^, ^11^

Guideline‐directed medical therapy is often initiated in specialist clinics but requires upward titration by a broader healthcare team with varying success.^6^, ^12^ People with HF are commonly hospitalized, and this event is an important opportunity to consider optimization of GDMT,^13^ given the high risk of readmission or death in the early months after discharge.^14^ Whilst early home visits or clinic follow‐up may help to optimize GDMT post‐discharge,^15^ changes to GDMT during hospitalization are also crucial. Indeed, the OPTIMIZE‐HF registry showed that people hospitalized for decompensation of HF who were continued on beta‐blocker therapy had lower mortality and rates of readmission following discharge.^16^ This has also been demonstrated with ACEi/ARB therapy, discontinuation of which was also associated with longer hospitalization, along with increased readmission and post‐discharge mortality.^17^

Recent studies have shown complex effects of HF hospitalization on GDMT titration, with upward and downward titration of GDMT both being common events.^9^ Moreover, other data show that people hospitalized for HF in the past 12 months are more likely to be receiving subtherapeutic doses of all GDMT.^11^ Notably, such trials focus on hospitalization due to decompensated HF, but people with HFrEF are more commonly hospitalized for other reasons, particularly non‐cardiovascular problems that may not be managed by cardiologists.^18^ Whether the cause of hospital admission is associated with unfavourable changes to GMDT has not been studied. The aim of this study is to characterize GDMT changes during hospital admission for patients with HFrEF stratified by cause of hospitalization, establish the reasons and risk factors for de‐escalation of GDMT, and address the prognostic impact of such changes.

## Methods

As described previously,^18^, ^19^, ^20^ a prospective, multi‐centre, observational cohort study recruited 1802 people with HFrEF to identify prognostic markers. This study was recruited in three phases, and this analysis is restricted to the third phase of 711 participants recruited between February 2012 and December 2014, which collected detailed hospitalization data.^18^ Inclusion criteria consisted of the presence of stable signs and symptoms of HF for >3 months in adults (≥18 years) with a left ventricular ejection fraction (LVEF) of ≤45% on transthoracic echocardiography. All participants were recruited from specialist HF clinics in four hospitals in West Yorkshire, UK. All participants provided informed written consent. The Leeds West Research Ethics Committee gave approval, and the study was conducted according to the principles expressed in the Declaration of Helsinki.

### Baseline assessment

Demographic and clinical data were collected at baseline. A past medical history of type 2 diabetes mellitus (T2DM), ischaemic heart disease (IHD), chronic obstructive pulmonary disease (COPD), or chronic kidney disease stage 4/5 (CKD 4/5) was determined at recruitment using medical records. A venous blood sample was taken for assessment of haemoglobin, electrolytes, creatinine, and albumin at baseline. Estimated glomerular filtration rate (eGFR) was calculated using the Modification of Diet in Renal Disease method.^21^ Functional status was assessed using the New York Heart Association (NYHA) classification. Resting 12 lead electrocardiograms (ECGs) were used to measure heart rate (HR) and QRS duration. Two‐dimensional transthoracic echocardiography was performed and reported by local cardiac physiologists, with LVEF calculated using Simpson's biplane method. In cases where imaging was suboptimal for Simpson's method, we relied on additional imaging with contrast enhanced ultrasound, cardiac magnetic resonance, or multiple‐gated acquisition scanning to assess LVEF.

### Ascertainment of hospitalization and mortality

All unplanned hospitalization episodes taking place before the censorship date of 18 February 2019 were recorded using electronic health records, as previously described.^18^ Institutional electronic health records were used to collect data including date of admission and discharge; vital signs on admission; lowest recorded eGFR; and highest recorded potassium (K+). The cause of hospitalization was defined by two independent investigators as being primarily attributed to one of the following major categories: (i) HF hospitalization; (ii) other cardiovascular hospitalization (e.g. arrhythmia or acute coronary syndrome, without decompensated HF); (iii) infection‐related hospitalization; and (iv) other non‐cardiovascular hospitalization (non‐cardiovascular cause excluding infection related). These definitions have previously been published.^18^ Admission and discharge doses of HFrEF GDMTs were determined from discharge letters (specifically for ACEi/ARBs, beta‐blockers, and MRAs; loop diuretic dose changes were also defined). Medications in each class were converted to the percentage of maximum licensed doses, and loop diuretics were converted to a furosemide daily dose equivalent, as previously published.^22^ The analysis was conducted before widespread use of angiotensin receptor neprilysin inhibitors (ARNIs) or SGLT2 inhibitors in the UK. All patients were registered with the UK Office of Population Censuses and Surveys, which provided details of time of death, with a final censorship date of 18 February 2019.

### Statistics

Statistical analyses were performed using SPSS v.27.0 (IBM). Continuous data are presented as mean [standard error of mean (SEM)] for normally distributed variables and median (25th to 75th centile) for non‐normally distributed variables. Categorical variables are presented as number (%). All statistical tests were two‐sided and considered significant at the 5% level. Normally distributed continuous data were compared using Student's *T* tests or ANOVA, and non‐normally distributed continuous data were compared using Mann–Whitney *U* tests or Kruskal–Wallis *H* tests. Categorical data were compared using Pearson's *χ* ^2^ tests. Univariate and multivariate predictors of GDMT dose reduction were derived using binary logistic regression analysis. Mortality analyses were performed using log‐rank tests and illustrated using Kaplan–Meier curves.

## Results

Of the 711 study participants, 467 were hospitalized at least once during a mean follow‐up period of 48.6 months. Characteristics of people who were hospitalized versus those not hospitalized are shown in Supporting Information, *Table* *S1*. Of these 467 participants, 40 died during hospitalization or were transferred to a palliative care facility and 13 had insufficient documentation of medication changes, resulting in 414 hospitalizations being included in this analysis, the characteristics of which are presented in *Table* *1*, according to the cause of hospitalization. Decompensated HF accounted for 13.3% of hospitalizations, other cardiovascular causes for 24.9%, other non‐cardiovascular for 38.6%, and infection for 23.2%. Participants with cardiovascular hospitalization (i.e. due to decompensated HF or other cardiovascular causes) were more likely to have a cardiac resynchronization therapy (CRT) or implantable cardioverter‐defibrillator (ICD) device *in situ*. Those admitted due to infection more commonly had COPD and had higher blood white cell count (WCC) at admission. Participants admitted with decompensated HF or infection had lower serum sodium, eGFR, and diastolic blood pressure (BP) compared with other causes of hospitalization.

**Table 1:** Patient characteristics according to cause of hospitalization

		HF hospitalization (*n* = 55)	Other CV hospitalization (*n* = 103)	Other non‐CV hospitalization (*n* = 160)	Infection hospitalization (*n* = 96)	*P*‐value
Age (years)		76 (68–81)	75 (65–81)	75.5 (67–82)	77 (70–82)	0.545
Female		14 (25.2%)	26 (25.2%)	49 (30.6%)	22 (22.9%)	0.55
NYHA class	I	3 (5.5%)	16 (15.5%)	21 (13.1%)	10 (10.4%)	0.498
	II	32 (58.2%)	56 (54.4%)	96 (60.0%)	55 (57.3%)	
	III	20 (36.4%)	31 (30.1%)	43 (26.9%)	30 (31.3%)	
	IV	0 (0.0%)	0 (0.0%)	0 (0.0%)	1 (1.0%)	
CRT/ICD		14 (25.5%)	27 (26.2%)	31 (19.4%)	9 (9.4%)	0.015
LVEF (%)		34 (25–40)	32 (26–40)	35 (25–40)	35 (24–40)	0.985
COPD		11 (20%)	13 (12.6%)	23 (14.4%)	32 (33.3%)	<0.001
DM		25 (45.5%)	39 (37.9%)	44 (27.5%)	37 (38.5%)	0.058
IHD		31 (56.4%)	68 (66.0%)	83 (51.9%)	55 (57.3%)	0.162
CKD		7 (12.7%)	10 (9.7%)	11 (6.9%)	13 (13.5%)	0.312
HR (b.p.m.)		73 (65–86)	74 (62–84)	75 (64–86)	79 (68–88)	0.329
Systolic BP (mmHg)		121 (112–135)	130 (110–145)	124 (110–140)	130 (110–138)	0.804
Diastolic BP (mmHg)		70 (60–76)	70 (60–80)	70 (60–80)	70 (60–80)	0.488
QRS interval (ms)		116 (102–148)	110 (96–142)	110 (96–142)	108 (95–137)	0.345
Haemoglobin (g/L)		130 (18)	132 (19)	131 (17)	132 (21)	0.751
Creatinine (μmol/L)		99 (75–129)	97 (82–144)	96 (79–125)	93 (78–131)	0.733
eGFR (mL/min/1.73 m^2^)		61 (39–75)	59 (39–75)	62 (45–82)	62 (39–85)	0.571
Serum Na+ (mmol/L)		139 (136–141)	140 (138–142)	140 (138–142)	140 (138–142)	0.291
Serum K+ (mmol/L)		4.4 (4.2–4.7)	4.5 (4.1–4.8)	4.5 (4.2–4.8)	4.45 (4.0–4.7)	0.668
Albumin (g/L)		42 (39–44)	43 (41–44)	42 (40–44)	41 (39–44)	0.018
*During admission*:						
WCC (×10^9^/L)		8.1 (6.2–10.1)	8.1 (6.4–9.8)	8.5 (6.6–10.8)	12.4 (8.7–16.6)	<0.001
Serum Na+ (mmol/L)		137 (133–140)	139 (137–141)	138 (136–140)	137 (133–140)	<0.001
Serum K+ (mmol/L)		4.5 (4.0–5.0)	4.4 (4.0–4.8)	4.5 (4.1–4.9)	4.45 (4.1–4.8)	0.294
Creatinine (μmol/L)		114 (90–190)	109 (89–156)	110 (81–176)	140 (95–214)	0.024
eGFR (mL/min/1.73 m^2^)		43 (29–69)	50 (37–67)	51 (29–73)	42 (25–58)	0.038
Systolic BP (mmHg)		123 (111–135)	128 (113–144)	125 (107–145)	120 (103–141)	0.228
Diastolic BP (mmHg)		69 (62–86)	73 (61–81)	71 (63–80)	67 (57–73)	0.017
HR (b.p.m.)		84 (63–98)	74 (63–84)	74 (65–88)	84 (65–92)	0.269
ACEi/ARB admission dose (% licensed max.)		25 (6.3–100)	50 (12.5–100)	50 (25–100)	50 (12.5–100)	0.135
ACEi/ARB discharge dose (% licensed max.)		25 (0–50)	33.3 (7.1–100)	50 (0–100)	25 (0–100)	0.662
Mean ACEi/ARB dose change (% licensed max.)		−6.7 (3)	−3.8 (2.1)	−13.4 (2.3)	−12.5 (2.4)	0.006^a^
Beta‐blocker admission dose (% licensed max.)		37.5 (12.5–75)	50 (25–100)	50 (25–100)	50 (18.8–75)	0.36
Beta‐blocker discharge dose (% licensed max.)		37.5 (25–75)	50 (25–100)	50 (25–87.5)	50 (12.5–75)	0.597
Mean beta‐blocker dose change (% licensed max.)		1.6 (2.6)	1.8 (2.3)	−3.6 (1.1)	0.3 (1.4)	0.001^a^
MRA at admission		24 (43.6%)	37 (35.9%)	61 (38.1%)	30 (31.3%)	0.469
MRA at discharge		37 (67.3%)	42 (40.8)	46 (28.8%)	28 (29.2%)	<0.001
Furosemide‐equivalent admission dose (mg/day)		40 (20–80)	40 (0–40)	40 (0–80)	40 (20–80)	0.058
Furosemide‐equivalent discharge dose (mg/day)		80 (80–160)	40 (0–80)	40 (0–40)	40 (20–80)	<0.001
Mean furosemide dose change (mg/day)		48.4 (7.8)	1.2 (2.9)	−3.5 (2.5)	3.3 (4.0)	<0.001^a^

ACEi/ARB, ACE inhibitor or angiotensin receptor blocker; BP, blood pressure; CKD, chronic kidney disease; COPD, chronic obstructive pulmonary disease; CRT/ICD, cardiac resynchronization therapy/implantable cardioverter‐defibrillator; CV, cardiovascular; DM, diabetes mellitus; eGFR, estimated glomerular filtration rate; HF, heart failure; HR, heart rate; IHD, ischaemic heart disease; K+, potassium; LVEF, left ventricular ejection fraction; MRA, mineralocorticoid receptor antagonist; Na+, sodium; NYHA, New York Heart Association; WCC, white cell count.

^a^ Denotes non‐parametric test presented with mean (standard error of mean) descriptive data.

### Prevalence and extent of guideline‐directed medical therapy dose changes

**Figure 1:**
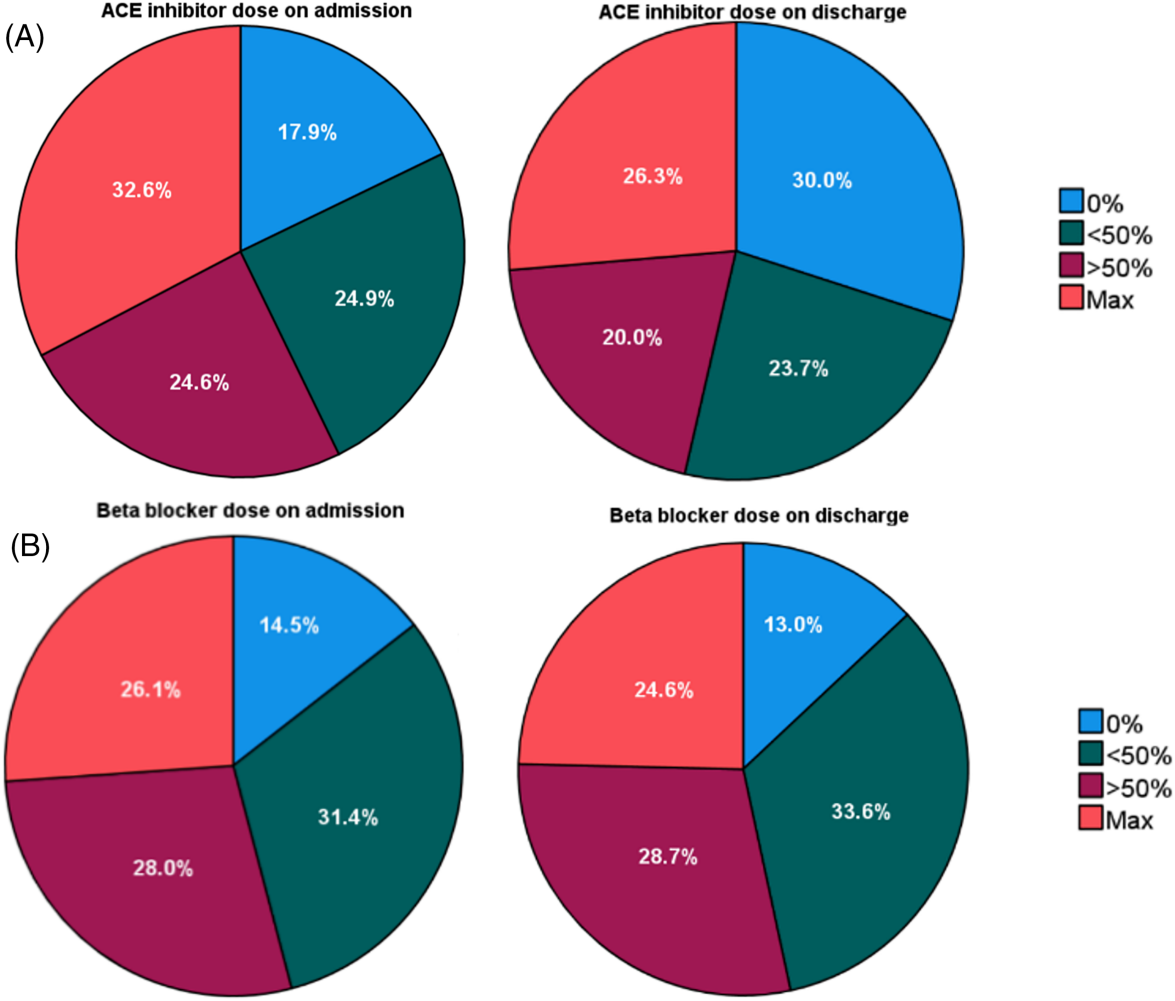
Use of ACEi/ARBs and beta‐blockers before and after hospitalization. Pie charts illustrate the dosage ranges of (A) ACEi/ARBs and (B) beta‐blockers at hospital admission (left) and discharge (right). Doses are expressed as percentage of maximum licensed dose. ACEi/ARBs, ACE inhibitors or angiotensin receptor blockers.

**Figure 2:**
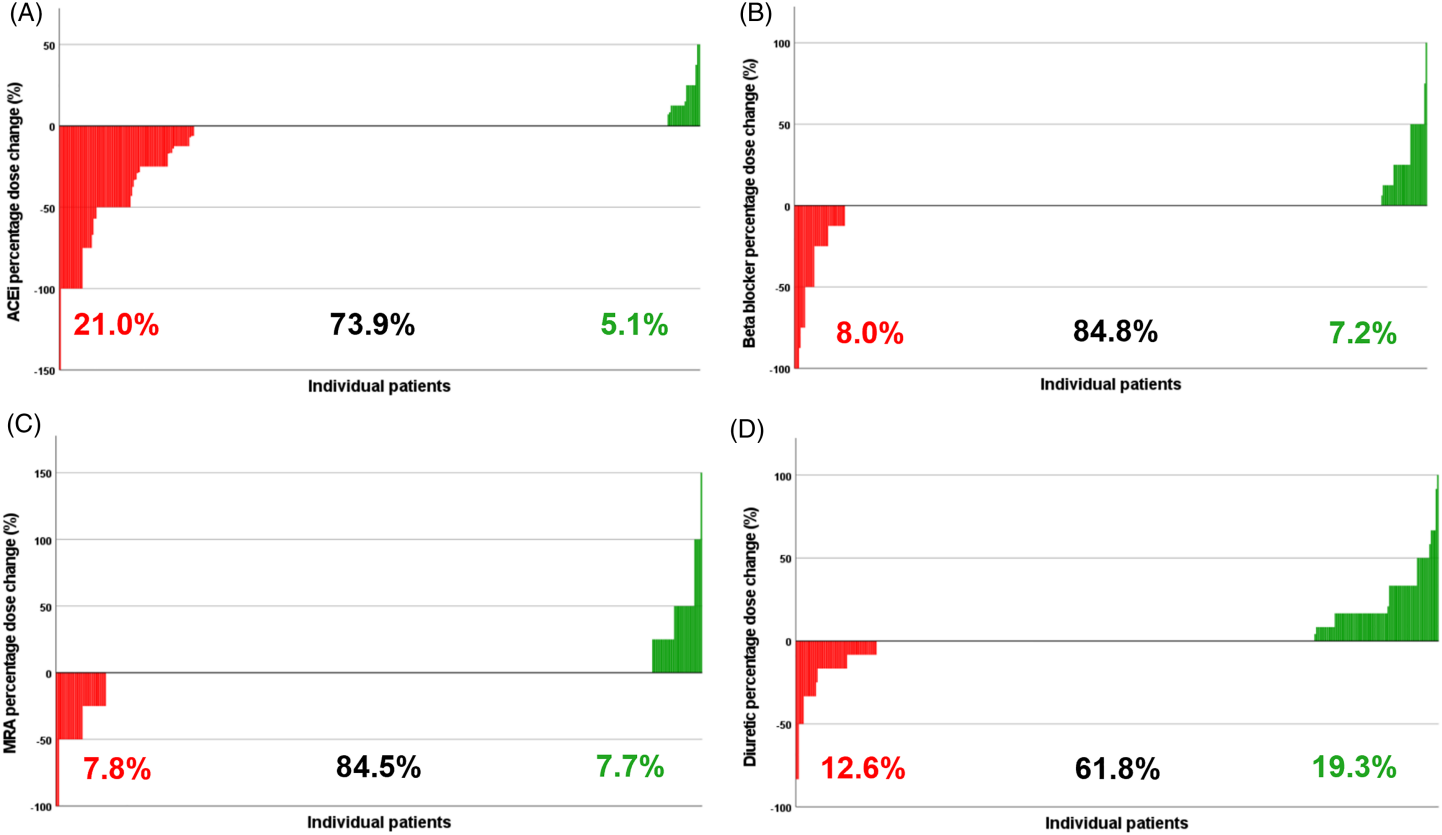
Prevalence and extent of guideline‐directed medical therapy dose change after hospitalization. Each bar represents individual patient % dose changes of (A) ACEi/ARBs, (B) beta‐blockers, (C) MRAs, and (D) loop diuretics from admission to discharge. Red bars show a dose decrease, green bars a dose increase, and no bar shows no change in dose. ACEi/ARBs, ACE inhibitors or angiotensin receptor blockers; MRAs, mineralocorticoid receptor antagonists.

The dose of ACEi/ARBs and beta‐blockers (expressed as percentage of maximum licensed dose), along with the use of MRAs and the dose of loop diuretics (expressed as furosemide‐equivalent dose per day), was assessed at admission and discharge from hospital (*Table* *1* and *Figure* *1*). At discharge, 26.3% of participants were receiving the maximum licensed dose of ACEi/ARB and 24.6% the maximum licensed dose of beta‐blockers. The percentage of participants receiving the maximum licensed dose of ACEi/ARB decreased by 6.3% during admission and 12.1% more people were not receiving any ACEi/ARB therapy at discharge. Participants receiving the maximum dose of beta‐blocker reduced by 1.5%, as did the percentage not receiving beta‐blocker. Only 37% of people were prescribed MRA therapy at discharge, whereas 73% received a loop diuretic. On discharge, 10% of people were receiving both the maximum dose of ACEi/ARB and beta‐blocker. Importantly, most hospitalizations were associated with no changes to GDMT or diuretic doses (*Figure* *2*). ACEi/ARBs were down titrated more commonly than other classes of medication, occurring in 21.0% of hospitalizations. Upward titration of GDMT was uncommon, whilst diuretics were increased in 19.3% of hospitalizations.

**Figure 3:**
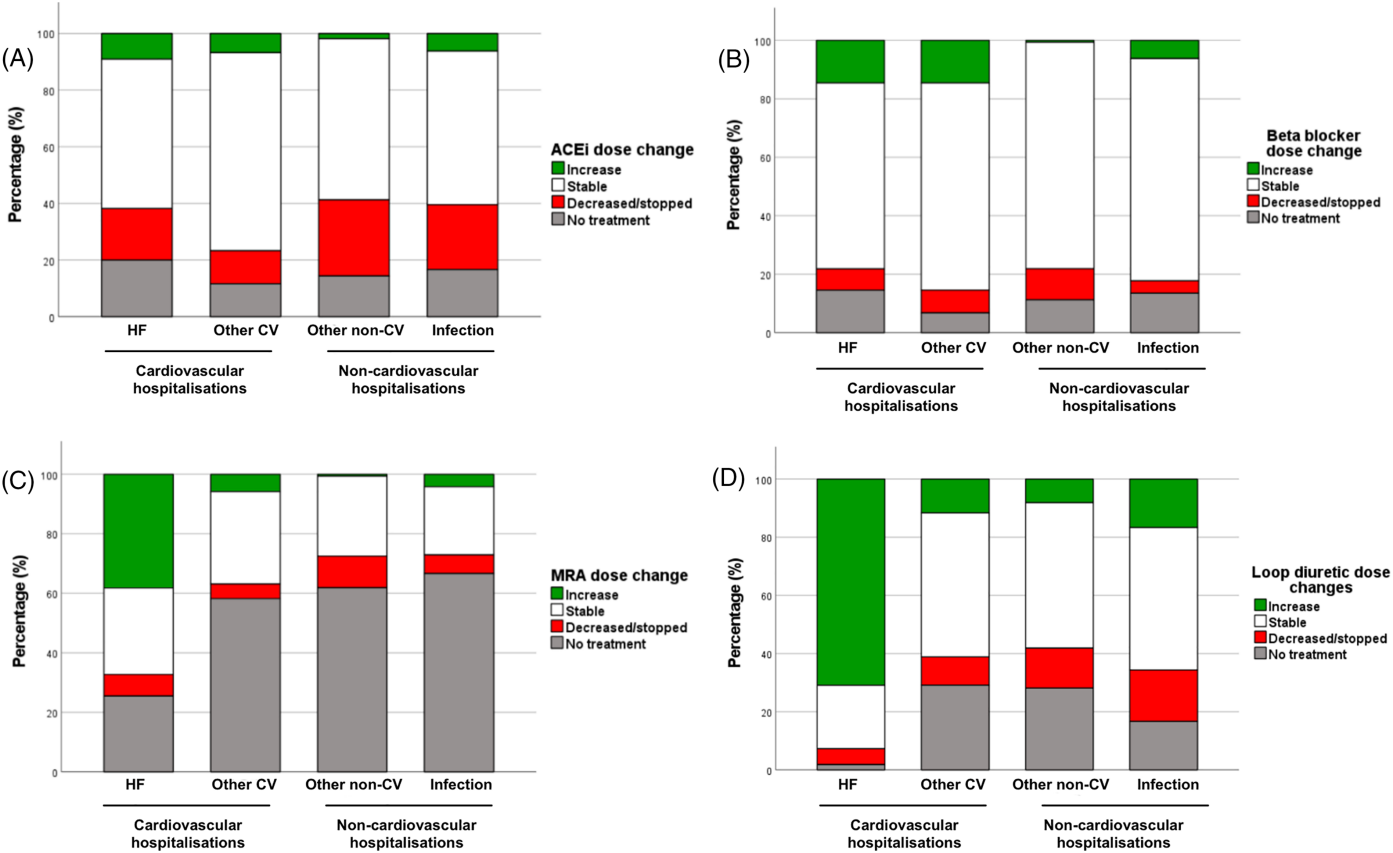
Guideline‐directed medical therapy dose changes according to cause of hospitalization. Stacked bars illustrate dose changes categories of (A) ACEi/ARBs, (B) beta‐blockers, (C) MRAs, and (D) loop diuretics during admission categorized according to the primary cause of admission. Red bars denote a dose decrease, green bars a dose increase, white bars no change in dose, and grey represents no treatment at admission or discharge. ACEi/ARBs, ACE inhibitors or angiotensin receptor blockers; CV, cardiovascular; HF, heart failure; MRAs, mineralocorticoid receptor antagonists.

ACEi/ARBs were more commonly down titrated during hospitalizations due to non‐cardiovascular than cardiovascular causes (25.4% vs. 13.9%; *P* = 0.005; *Figure* *3* *A*). However, looking in more detail at cardiovascular hospitalizations, ACEi/ARB were decreased in 18.2% of people with decompensated HF and only escalated in 9.1%; indeed, 29.1% of this group were not discharged on any ACEi/ARB. Beta‐blockers were more commonly up titrated in cardiovascular rather than non‐cardiovascular admissions (14.6% vs. 2.7%; *P* < 0.001; *Figure* *3* *B*), with no differences in the proportion undergoing down titration between these groups. Up titration of MRAs and loop diuretics was mainly performed during admissions for decompensated HF (*Figure* *3* *C*,*D*), occurring in 38.2% and 70.9%, respectively. Loop diuretics were more commonly down titrated in non‐cardiovascular than cardiovascular hospitalizations (15.2% vs. 8.2%; *P* = 0.037).

### Risk factors for guideline‐directed medical therapy dose reduction

Reasons for ACEi/ARB dose changes were well documented in discharge letters, with dose reductions being due to deteriorating kidney function in 55.2% (*n* = 48), hypotension in 37.9% (*n* = 33), and hyperkalaemia in 16.1% (*n* = 14). A combination of these factors often contributed to down titration, and each reason was recorded separately in our subsequent analyses. Reasons for down titration of beta‐blockers were poorly recorded in discharge letters with 42.4% of reductions having no justification. Hypotension (45.5%, *n* = 15) and bradycardia (21.2%, *n* = 7) were the most common reasons for dose reduction. Notably, ACEi/ARB dose reduction was associated with longer hospital stay [10 (4–19) vs. 4 (2–8) days; *P* < 0.001], as was beta‐blocker dose reduction [10 (4.5–18) vs. 4 (2–9) days; *P* = 0.001].

Univariate binary logistic regression was performed to define factors associated with reduction of ACEi/ARB dose (*Table* *2*) and revealed statistically significant associations with older age and lower LVEF, recorded at study recruitment. At the point of hospitalization, other factors significantly associated with ACEi/ARB dose reduction were lower serum sodium, higher serum potassium, lower eGFR, lower systolic BP, a non‐cardiovascular cause of hospitalization, and not receiving care from a cardiologist. Multivariate analysis including these factors (*Table* *2*) demonstrated statistically significant independent contributions from LVEF, eGFR, serum potassium, systolic BP, and a non‐cardiovascular cause of hospitalization. The only significant univariate association with beta‐blocker dose reduction was eGFR on admission [odds ratio 0.984 (0.969–1.000); *P* = 0.048].

**Table 2:** Factors associated with ACEi/ARB dose reduction during hospitalization

	Univariate analysis		Multivariate analysis		
	Odds ratio (95% CI)	*P*‐value	Odds ratio (95% CI)	*P*‐value
Age (per year)	1.026 (1.004–1.048)	0.018	1.011 (0.979–1.043)	0.508
LVEF (per %)	0.974 (0.952–0.988)	0.034	0.959 (0.925–0.994)	0.021
*On admission*:				
Serum Na+ (per mmol/L)	0.89 (0.85–0.94)	<0.001	0.94 (0.87–1.01)	0.089
Serum K+ (per mmol/L)	3.31 (2.29–4.78)	<0.001	2.35 (1.39–3.97)	0.001
eGFR (per mL/min/1.73 m^2^)	0.964 (0.953–0.976)	<0.001	0.968 (0.950–0.985)	<0.001
Systolic BP (per mmHg)	0.981 (0.969–0.992)	0.001	0.988 (0.976–1.000)	0.046
Non‐CV admission	2.11 (1.21–3.58)	0.006	3.20 (1.25–8.18)	0.015
Treated by cardiologist	0.58 (0.34–0.99)	0.048	1.05 (0.41–2.70)	0.92

BP, blood pressure; CI, confidence interval; CV, cardiovascular; eGFR, estimated glomerular filtration rate; K+, potassium; LVEF, left ventricular ejection fraction; Na+, sodium.

Logistic regression analysis presenting baseline and admission factors associated with ACE inhibitor or angiotensin receptor blocker (ACEi/ARB) dose reduction. In the multivariate analysis, all presented covariates were simultaneously included in the model.

### Prognostic implications of guideline‐directed medical therapy dose reduction

**Figure 4:**
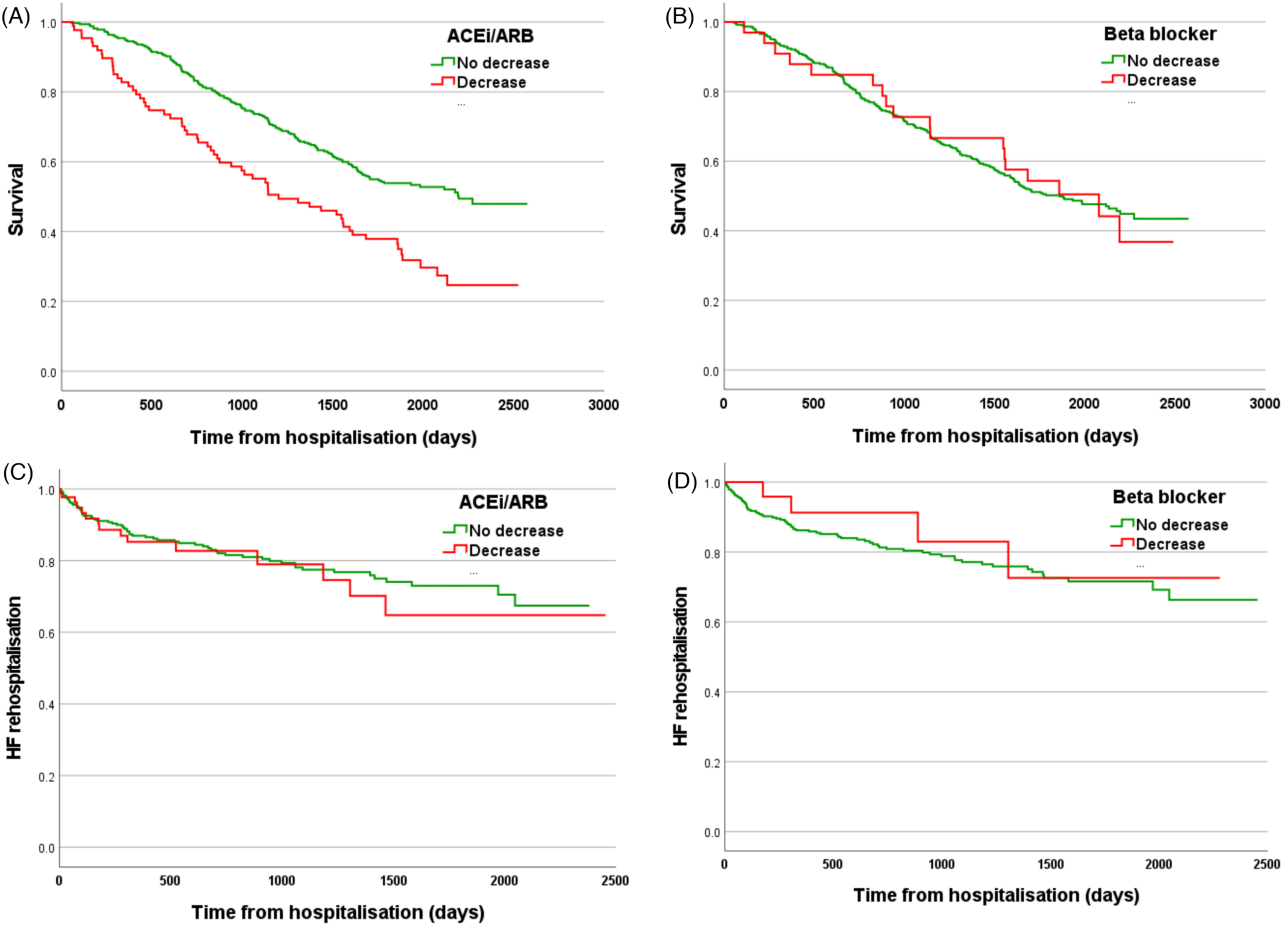
Outcomes associated with guideline‐directed medical therapy dose reduction. Kaplan–Meier survival curves showing the association between ACEi/ARB or beta‐blocker dose reduction and mortality or re‐hospitalization due to decompensated HF. ACEi/ARB, ACE inhibitor or angiotensin receptor blocker; HF, heart failure.

Kaplan–Meier mortality curves for participants with or without ACEi/ARB dose reduction during hospitalization illustrate markedly worse post‐discharge survival in those with any dose reduction (*Figure* *4* *A*; *P* < 0.001 by log‐rank test). Cox regression analysis revealed that accounting for age did not alter the mortality risk associated with ACEi/ARB reduction [hazard ratio 1.75 (95% confidence interval 1.30–2.35); *P* < 0.001]. However, further addition of LVEF, admission eGFR, admission serum potassium, admission systolic BP, and cardiovascular/non‐cardiovascular cause of hospitalization (because these were independently associated with dose reduction) resulted in loss of the mortality risk associated with ACEi/ARB reduction [1.34 (0.85–2.12); *P* = 0.21]. Beta‐blocker dose reduction was not associated with any reduction in post‐discharge survival (*Figure* *4* *B*). In terms of re‐hospitalization due to decompensated HF, neither ACEi/ARB nor beta‐blocker dose reduction was associated with increased risk (*Figure* *4* *C*,*D*).

## Discussion

Our detailed analysis of hospitalization in people with HFrEF has led to a number of important findings, adding to the evidence base that hospitalizations are not only a missed opportunity to optimize GDMT but also often associated with de‐escalation of prognosis improving therapy. First, we show that over 70% of hospitalizations are associated with no change in GDMT, even though over half of hospitalized participants were at sub‐maximal doses, illustrating the scale of therapeutic inertia. Second, we show that over one in five people leave hospital taking a lower dose of ACEi/ARB, with even 18% of decompensated HF admissions resulting in ACEi/ARB dose reduction. Third, beyond the expected associations of ACEi/ARB de‐escalation with admission renal impairment, hyperkalaemia, and hypotension, we show that lower LVEF, non‐cardiovascular hospitalization, and care from non‐cardiologists are risk factors for this, with non‐cardiovascular hospitalization independently associated with dose reduction. Finally, we show that ACEi/ARB dose reduction, but not beta‐blocker dose reduction, is associated with markedly worse post‐discharge survival, although without differences in re‐hospitalization due to decompensated HF. Overall, our data suggest that there remains a pressing need to improve GDMT optimization during and immediately after hospitalization and not only for people hospitalized due to decompensated HF.

Hospitalization is an important opportunity to escalate HF medications under close supervision and monitoring, but it is also a complex period when the priority may change from long‐term to short‐term survival, often due to factors other than decompensated HF. Indeed, reduction of HFrEF GDMT during admission may be unavoidable in some instances, and we noted clear documentation of important reasons to temporarily reduce therapy, such as acute kidney injury, hyperkalaemia, and symptomatic hypotension; dose reductions were also associated with longer duration of hospitalization. This aligned with our logistic regression analysis of risk factors for ACEi/ARBs dose reduction, which also implicated older age and lower LVEF. Notably, a recent systematic review of sub‐target dosing of ACEi/ARBs identified many of the same risk factors across heterogeneous populations with HFrEF,^23^ although our multivariate analysis suggests that only some are independently associated with this phenomenon.

ACEi/ARB dose reduction was linked to a substantial and early increase in age‐adjusted mortality after discharge, which lost statistical significance after accounting for acute renal impairment, hyperkalaemia, hypotension, and the cause of hospitalization. Unfortunately, we lack data on how frequently and rapidly abnormal renal function, electrolytes, and BP normalized after discharge, and whether this was associated with re‐escalation of medical therapy. However, it seems likely that a significant proportion of people would be candidates for re‐escalation of ACEi/ARB soon after discharge, although the inertia we observed during hospitalization would suggest that such optimization is probably uncommon. Indeed, intensive support after decompensated HF hospitalization has recently been shown to achieve only modest improvements in GDMT dosing, which did not translate into improved hard outcomes.^24^ Whilst we did not see increased risk of re‐hospitalization with HF in people with reduced ACEi/ARB or beta‐blocker dose, the observed 1 year risk exceeded 10%, serving as a reminder of the scope for escalation of GDMT to improve this, especially in the ARNI and SGLT2 inhibitor era.^25^ The absence of increased risk of re‐hospitalization with HF after ACEi/ARB dose reduction, in the context of an increased mortality rate, is also notable. We speculate that this may reflect generally poorer health in people requiring ACEi/ARB dose reduction, perhaps leading to death from other causes before HF decompensates or leading to decisions to take a palliative approach aiming to avoid hospitalization. Further studies will be required to explore these possibilities.

Regarding beta‐blockers, the rationale for dose reduction was poorly documented, which is likely to hinder attempts to re‐escalate beta‐blocker dose post‐discharge. However, our analysis suggests that current practice in beta‐blocker dose reduction during hospitalization is not linked to worse prognosis, so this may suggest that these actions were appropriate and that efforts to optimize GDMT post‐discharge should prioritize ACEi/ARBs.

Whilst our analysis has produced some valuable insights, it is important to set out its limitations. First, our observational data cannot be used to infer causality, and therefore, it is unclear how much of the adverse prognosis linked with ACEi/ARB dose reduction reflects confounding from factors such as comorbidity, versus direct impacts of changes to ACEi/ARB. Second, our analysis is relatively small and restricted to UK practice prior to the use of ARNI and SGLT2 inhibitors, meaning our findings may not generalize to other healthcare systems or eras. However, the increasing complexity of HFrEF medical therapy, along with the rising multimorbidity of people with HFrEF,^1^ makes it likely that hospitalization will be increasingly associated with adverse changes to GDMT dosing. Third, we do not have data on serum N‐terminal pro‐brain natriuretic peptide (NT‐proBNP) or clinical signs of congestion at hospital admission or discharge, given the predominance of non‐cardiovascular hospitalization in our study. However, such data would be useful in clarifying in more detail the association between non‐cardiovascular hospitalization and change in cardiovascular status.

In conclusion, this is the first study to identify and quantify the changes that are made to HFrEF GDMT during hospitalization stratified by its principal cause. In the majority of hospitalizations, including those due to decompensated HF, no change was made to GDMT. ACEi/ARBs were the most common therapy to be de‐escalated, and this was more common in non‐cardiovascular hospitalizations, especially in the context of low BP, impaired renal function, hyperkalaemia, and care from a non‐cardiologist. Reduction of ACEi/ARB dose was associated with worse age‐adjusted post‐discharge survival. These observations suggest that greater attention needs to be placed on GDMT optimization during and immediately after hospitalization, particularly in people admitted due to non‐cardiovascular problems.

## Conflict of interest

V.K.G. has received honoraria from Novartis, Abbott, Astra Zeneca, and Boehringer Ingelheim. A.S. (Anshuman Sengupta) has received speaker fees from Pfizer. M.T.K. has received speaker fees from Merck and Novo Nordisk and unrestricted research awards from Medtronic. K.K.W. has undertaken consultancy work for Medtronic and Cardiac Dimensions; acted as an investigator and steering committee member for studies co‐ordinated by Novartis and Medtronic; and has received speaker fees from Cardiac Dimensions, Medtronic, Microport, Abbott, Pfizer, Bayer, and BMS.

## Funding

This work was funded by the British Heart Foundation (PG/08/020/24617).

## Supporting information


**Table S1.** Baseline characteristics of hospitalised patients.
