# Supplementary material for: Reduction of heart failure guideline‐directed medication during hospitalization: prevalence, risk factors, and outcomes
Source: ESC Heart Fail. 2022 Jul 7;9(5):3298–307. doi: 10.1002/ehf2.14051 (PMC9715809; doi:10.1002/ehf2.14051)
Supplement: Supplementary file 1 — Table S1. Baseline characteristics of hospitalised patients. [file EHF2-9-3298-s001.docx]

**Appendix Table 1: Baseline characteristics of hospitalised patients**

|  | | **Total**  **(n=711)** | **Not hospitalised (n=244)** |  | **Hospitalised (n=467)** | **p-value** |
| --- | --- | --- | --- | --- | --- | --- |
| Age (years) |  | 75 (65-80) | 70 (60-78) |  | 76 (68-82) | <0.001 |
| Female |  | 195 (27.4%) | 65 (26.6%) |  | 130 (27.8%) | 0.734 |
| NYHA class | I | 107 (15.0%) | 50 (20.5%) |  | 57 (12.2%) | 0.002 |
|  | II | 408 (57.4%) | 144 (59.0%) |  | 264 (56.6%) |  |
|  | III | 192 (27.0%) | 48 (19.7%) |  | 144 (30.8%) |  |
|  | IV | 4 (0.6%) | 2 (0.8%) |  | 2 (0.4%) |  |
| CRT/ICD |  | 153 (21.5%) | 64 (26.2%) |  | 89 (19.1%) | 0.027 |
| LVEF (%) |  | 33 (24-40) | 32 (24-40) |  | 34 (25-40) | 0.235 |
| COPD |  | 116 (16.3%) | 27 (11.1%) |  | 89 (19.1%) | 0.006 |
| DM |  | 224 (31.5%) | 61 (25.0%) |  | 163 (34.9%) | 0.007 |
| IHD |  | 379 (53.3%) | 110 (45.1%) |  | 269 (57.6%) | 0.001 |
| CKD |  | 62 (8.7%) | 13 (5.3%) |  | 49 (10.5%) | 0.02 |
| HR (bpm) |  | 75 (64-86) | 73.5 (64-87) |  | 75 (64-86) | 0.816 |
| Systolic BP (mmHg) | | 123 (110-140) | 120 (110-140) |  | 124 (110-140) | 0.397 |
| Diastolic BP (mmHg) | | 70 (60-80) | 70 (64-80) |  | 70 (60-80) | 0.027 |
| QRS interval (ms) | | 112 (98-146) | 117.5 (98-150) |  | 110 (96-142) | 0.018 |
| Haemoglobin (g/L) | | 133.0 (18.5) | 138.7 (17.1) |  | 130.5 (18.7) | <0.001 |
| Creatinine (umol/L) | | 95 (75-126) | 87 (72-109.5) |  | 97 (78-134) | <0.001 |
| eGFR (mL/min/1.73m²) | | 64 (45-81) | 69 (53.5-86) |  | 59 (40-79) | <0.001 |
| Serum Na+ (mmol/L) | | 140 (138-142) | 140 (139-142) |  | 140 (138-142) | 0.095 |
| Serum K+ (mmol/L) | | 4.4 (4.2-4.8) | 4.4 (4.2-4.7) |  | 4.5 (4.2-4.8) | 0.245 |
| Albumin (g/L) | | 43 (40-45) | 44 (42-46) |  | 42 (40-44) | <0.001 |

NYHA, New York Heart association; CRT/ICD, cardiac resynchronisation therapy/ implantable cardiac defibrillator; LVEF, left ventricular ejection fraction; COPD, chronic obstructive pulmonary disease; DM, diabetes mellitus; IHD, ischaemic heart disease; CKD, chronic kidney disease; HR, heart rate; BP, blood pressure; eGFR, estimated glomerular filtration rate, Na+, sodium; K+, potassium. * denotes non-parametric test presented with mean (SEM) descriptive data
